# Supplementary material for: A comparison of presentations and outcomes of salvage versus non-salvage abdominal free flap breast reconstructions—Results of a 15-year tertiary referral centre review
Source: PLoS One. 2023 Nov 1;18(11):e0288364. doi: 10.1371/journal.pone.0288364 (PMC10619878; doi:10.1371/journal.pone.0288364)
Supplement: Attachment — Submitted filename: Response to reviewers.docx [file pone.0288364.s002.docx]

|  | **Box 186**  **Addenbrooke’s Hospital,**  **Hills Road,**  **Cambridge**  **CB2 0QQ**  Tel +44 01223 256 284  Fax +44 01223 257 177 |
| --- | --- |
|   Department of Plastic & Reconstructive Surgery |  |
|  |  |

Dr Emily Chenette

Editor In Chief at Public Library of Science (PLOS)

Dated March 30^th^, 2023

Dear Dr Emily Chenette,

**Re: A comparison of presentations and outcomes of salvage versus non-salvage abdominal free flap breast reconstructions – results of a 15-year tertiary referral centre review**

We are hereby submitting our revised manuscript. We would like to thank the reviewers’ for their insightful comments. We have revised our manuscript taking into account the reviewers’ comments and highlight our responses below. We believe the comments have helped improve the manuscript significantly and you will find it suitable for publication in the present format.

Kind Regards,

Dr Christine Bojanic, BSc MBBS MRSB MPhil(Cantab)

on behalf of Professor Charles M Malata BSc, MB ChB, MRCS, LRCP, FRCS(Glasg), FRCS(Plast)

Visiting Professor of Anglia Ruskin University School of Medicine, Cambridge & Chelmsford

Consultant Plastic Surgeon, Cambridge University Hospitals NHS Foundation Trust, Addenbrooke’s Hospital. Tel: +44 1223 586672. E-mail: [cmalata@hotmail.com](mailto:cmalata@hotmail.com)

RESPONSES TO REVIEWERS’ COMMENTS

| Reviewers’ Comments | Authors’ Response |
| --- | --- |
| Reviewer #1:  I would like to commend the authors for their interesting manuscript which provides insight on a peculiar side of breast reconstruction, by sharing their experience as a tertiary referral center. However, the manuscript presents aspects which warrant some revisions. They have been subdivided into sections according to the areas of concern. | Reply to Reviewer #1:  Many thanks for your insightful and informative comments.  We thoroughly appreciate your input. Thank you for subdividing your comments by section for ease. We have endeavoured to respond to each comment in turn, these changes have been highlighted. |
| Reviewer #1:  Abstract: - Abstracts should preferably not include abbreviations or acronyms and have a word limit of 300 words for this journal. Your current abstract counts 255 words. Please consider replacing the acronyms with the fully expanded terms. | Reply to Reviewer #1:  We have replaced all acronyms in the abstract. |
| Reviewer #1:  - The study compared 41 salvage flaps to 42 immediate or delayed flaps for breast reconstruction. Please specify in the “Methods” section of your abstract that all featured patients received an abdominally-based free flap. | Reply to Reviewer #1:  The new manuscript abstract reflects this change, thank you for your helpful comment. |
| Reviewer #1:  - What types of outcomes were assessed? From your description it would seem that you assessed clinical outcomes with operative times, intraoperative blood loss and complication rates. The aim of your study should be stated more clearly in the introductory section of the abstract where you state the aim of your study. Additionally, state the nature of the study you conducted more explicitly. | Reply to Reviewer #1:  The abstract has been altered accordingly. |
| Reviewer #1:  Introduction: - I acknowledge your definition of “tertiary” or “salvage breast reconstruction” which of course can be traced back to Hamdi et al.’s paper from 2010 (ref. 13). This concern is purely on a semantic standpoint: some consider “secondary breast reconstruction” as the “reconstructive procedure to correct complications and to improve the aesthetics when a patient is dissatisfied with her initial reconstruction” (Hee Chang Ahn et al. in J Korean Soc Plast Reconstr Surg. 2009), as opposed to primary reconstructions. Others interchangeably use “primary” as “immediate”, and “secondary” as “delayed” procedures, as did Hamdi et al. in their 2010 paper. My best recommendation is to either clear the definitions you chose in the introduction of your manuscript, or to scrape “primary”, “secondary” and “tertiary” altogether (including from the title), referencing the confusion in definitions, and just use “salvage” and “non-salvage” (i.e. immediate and delayed). | Reply to Reviewer #1:  We agree with the reviewer’s request to be clearer in terms of definitions. We have therefore removed the word tertiary except at one place in the discussion to refer to the original description by Moustapha Hamdi. |
| Reviewer #1:  - The introduction is otherwise well-thought and eases into the subject appropriately. However, consider implementing the following information to strengthen your concepts: o Breast implant lifespan has been estimated around 10 years, with patients potentially undergoing revisional surgeries as often as 4 times or more in their lifetime (PMID: 36376583). o Regarding silicone extravasation, recent evidence found breast implant ruptures to be extracapsular in many as 25.8% of cases (PMID: 36229658). | Reply to Reviewer #1:  Many thanks indeed for this comment – as you rightly suggest referring to the breast implant life span and the type of implant ruptures would help strengthen our paper’s message.  However, our patients did not undergo surgery for impaired implant integrity (i.e., reduced lifespan) but rather pain, poor cosmesis and severe capsular contracture.  We have incorporated the high risk of extracapsular ruptures in the introduction. |
| Reviewer #1:  Material and Methods: - Please clarify whether the study you conducted is “simply” a retrospective chart review or specifically a case-control study. Regardless of which, ensure that your paper is prepared in accordance with STROBE guidelines. | Reply to Reviewer #1:  This was a retrospective matched cohort study (not a case-control study) and the STROBE guidelines were kept in mind when preparing the manuscript and during the study conduct. |
| Reviewer #1:  - It appears that the patients from the non-salvage group were chosen chronologically. Were they still matched to the salvage group according to patient demographics (i.e. age and BMI)? It would seem that way as suggested by the p-values in table 1 showing that the differences between groups were not statistically relevant. This has not been explicitly stated in your manuscript, and should be addressed. | Reply to Reviewer #1:  We only matched patients chronologically. There were no additional selection criteria, and any similarities/differences are, as you rightly pointed out, not statistically relevant.  We have stated this in the manuscript. |
| Reviewer #1:  - The justification of why you only included procedures performed by the senior author to exclude the effects of learning curve (“Due to the long timeframe of the study […] selection bias.” In Materials and methods, “Patient sample” subheading, p. 4, lines 100-102) has no place in this portion of the manuscript and should be moved in the discussion. Additionally, your study features bilateral cases as well. Was a 2-team approach used for those cases or were they also performed by a single surgeon? If a second surgeon was used, would you say that the difference in experience between the senior surgeon and a hypothetical second surgeon could have affected the results in this group due to a difference in learning curve? | Reply to Reviewer #1:  The patient sample subheading has been removed as per the reviewer’s suggestion.  For the bilateral cases the “2^nd^ surgeon” was a resident or fellow who carried out the exposure of recipient internal mammary vessels and on occasion part raised the flap. However apart from these assistants the operations were carried out by the senior surgeon. The trainees rotate to the breast reconstruction firm every 6 months. The senior author performed all the operations otherwise. |
| Reviewer #1:  - Additionally, inclusion and exclusion criteria should be listed more clearly and explicitly. | Reply to Reviewer #1:  Thank you for your comment. We have amended the manuscript to include your helpful comment. There was only one inclusion criterion which was all patients operated on by the senior surgeon for salvage of failed prosthetic reconstructions or prosthetic reconstructions with recalcitrant complications necessitating salvage surgery. None of the patients identified were excluded. |
| Reviewer #1:  - Consider changing your terminology in the manuscript as well as in Table 3, by replacing “partial flap necrosis” with “fat necrosis” according to the Rao grading system for consistency (PMID: 25415090). | Reply to Reviewer #1:  Thank you for your comment, this is reflected within Table 3. |
| Reviewer #1:  Discussion: - Your salvage and non-salvage groups were matched according to age and BMI alone. Why could they not be matched according to their comorbidities, mastectomy type and donor site characteristics as well? Please address this as a potential limitation. | Reply to Reviewer #1:  The groups were matched by chronology alone, and any other matched factors were purely coincidental.  We accept this potential limitation however, there were no differences in co-morbidities. The patients could not be matched for mastectomy type as the principal aim of the study was to address the outcomes of the tertiary reconstructions and the mastectomies in these patients had been carried out years ago. |
| Reviewer #1:  - You reported no case of either partial flap loss or fat necrosis in your study. While this is impressive, consider addressing some of the identified predictive and protective factors for partial necrosis in DIEP flap breast reconstruction (PMID: 23851375). | Reply to Reviewer #1:  Santanelli et al have addressed this subject in their publications but it was not possible in a retrospective study to address these factors such as nulliparity and type of intravenous fluids administered as these was not recorded in the patients’ notes. We have added a sentence in our discussion to reflect that this would be an important consideration to take into account in future studies. We have also cited the paper you quote here.  Similarly, about half of the flaps were performed before we had access to CT angiography to determine whether the better vessels were the medial row or lateral row perforators. There was no statistically significant difference in the number of smokers, a known risk factor for fat necrosis. |
| Reviewer #1:  - Most patients received a DIEP flap. It would be interesting for the authors to briefly share their reconstructive ladder in terms of salvage options according to patient characteristics (i.e. breast size volume, donor site characteristics and laterality). | Reply to Reviewer #1:  The flap choice was based on the tissue availability. A decision was made to use abdominal flaps rather than secondary flaps for salvage because of their robustness and ready availability of reliable well vascularized tissue. This study was therefore only restricted to abdominal flaps.  Notwithstanding this, the senior author does not set out to perform an SIEA flap. Whether the patient ends up with an SIEA flap is an intraoperative decision based on the size and pulsation of the artery and whether half or less than half the abdominal pannus is required to match the size of the breast. The senior author favours the use of the contralateral flap for better flap inset whether the reconstruction is unilateral or bilateral. Bipedicled flaps were used when the volume of the flap that can be reliably carried on one vascular pedicle exceeded the target breast size. Muscle-sparing free TRAM flaps were only used if, during DIEP flap harvest, the perforators were deemed to be of insufficient / inadequate calibre and so some muscle was included around the perforator (s) to protect them and thus improve the viability of the flap. There was no plan to raise an MS-free TRAM preoperatively. None of the patients underwent non-abdominal free flaps for salvage. Therefore the selection of the flap falls outside the remit of this paper. |
| Reviewer #1:  Post-mastectomy radiotherapy has indeed been found to cause complications following beast reconstruction, with a study from Carnevale et al. reporting major complications and the need to reoperate in 7% of their 46-patient cohort (PMID: 23801395). Would you say that this complication rate is higher in your institution? Could that be biased by the fact that yours is a tertiary referral center? Please address this in your discussion. | Reply to Reviewer #1:  Thank you very much for raising this important point regarding PMRT. Interestingly almost 90% of the salvage cohort had had post-mastectomy radiotherapy versus only a quarter of the non-salvage cases. Our return to theatre was comparable to the data reported by the group from La Sapienza. It is true that the major complications were higher at 17% and 24% but this may reflect the small patient numbers in our series thus giving high percentages. We also tend to see more of the difficult cases who have been refused surgery elsewhere being referred to our tertiary referral center. |
| Reviewer #1:  - When assessing clinical outcomes of your abdominally-based free flaps, were time- dependent factors in DIEP flap breast reconstruction taken into consideration? Is has been demonstrated that increased flap weight, related perforators number, and venous drainage negatively influence operative time (PMID: 28758229). | Reply to Reviewer #1:  No, these factors were not taken into consideration as in a retrospective study the number of perforators was inconsistently recorded and there was no comment in the operation notes on the predominant venous drainage (the superficial system or the deep system). There was no significant difference in the flap weights of the two cohorts. The ischaemia time did not differ significantly. |
| Reviewer #1:  - The authors discuss using the internal mammary vessels as recipient vessels, which at times can be damaged from radiotherapy. Could having used recipient vessels in the axilla been a viable alternative if not even the recipient area of choice especially in these cases? (PMID: 24782202). | Reply to Reviewer #1:  We did not encounter any damage to the internal mammary vessels from radiotherapy. This might be a reflection of different radiotherapy regimens in different countries/ continents. The IMVs are our preferred recipients for reasons that are well documented in the literature. We did not resort to the use of the subscapular-thoracodorsal system as we prefer to leave these as a backup in case the patient needed a latissimus dorsi flap in case of flap loss. Fortunately, all our flaps were successful. Theoretically the axilla could be an alternative recipient site, but it could on occasions have also been irradiated or the vessels could have been damaged by previous axillary clearance. The circumflex scapular vessels are however “immune” from radiotherapy and surgical damage but suffer from challenging microsurgical position (during performance of the microanastomoses) and can compromise optimal flap inset as flaps can be more lateral than ideal because of the CSV pedicle’s short length. |
| Reviewer #1:  - This study did not account for type and volume of breast implants originally placed in patients from the salvage group. Consider addressing this as a possible shortcoming to your study and reference this study from Zhao et al. which found larger-sized implants to be more commonly associated with salvage reconstruction and conversion to abdominally- based free flaps (PMID: 29734445). | Reply to Reviewer #1:  This is an important parameter to account for, thank you for your observation. The implant sizes were not recorded and some of them were ruptured rendering any attempted assessment of implant volume and its relation to the success of the salvage reconstruction inaccurate. |
